# DNMT and HDAC inhibitors modulate MMP-9-dependent H3 N-terminal tail proteolysis and osteoclastogenesis

**DOI:** 10.1186/s13072-019-0270-0

**Published:** 2019-04-16

**Authors:** Yonghwan Shin, Nikhil B. Ghate, Byoungsan Moon, Kyungpyo Park, Wange Lu, Woojin An

**Affiliations:** 1 Department of Biochemistry and Molecular Medicine, Norris Comprehensive Cancer Center, University of Southern California, Los Angeles, CA 90089 USA; 2 Department of Stem Cell Biology and Regenerative Medicine, Broad Center for Regenerative Medicine and Stem Cell Research, University of Southern California, Los Angeles, CA 90089 USA; 3 Department of Physiology, School of Dentistry, Seoul National University, Seoul, 110-749 Korea

**Keywords:** MMP-9, H3 proteolysis, DNA methylation, Osteoclast differentiation, 5-Aza-dC, TSA

## Abstract

**Background:**

MMP-9-dependent proteolysis of histone H3 N-terminal tail (H3NT) is an important mechanism for activation of gene expression during osteoclast differentiation. Like other enzymes targeting their substrates within chromatin structure, MMP-9 enzymatic activity toward H3NT is tightly controlled by histone modifications such as H3K18 acetylation (H3K18ac) and H3K27 monomethylation (H3K27me1). Growing evidence indicates that DNA methylation is another epigenetic mechanism controlling osteoclastogenesis, but whether DNA methylation is also critical for regulating MMP-9-dependent H3NT proteolysis and gene expression remains unknown.

**Results:**

We show here that treating RANKL-induced osteoclast progenitor (OCP) cells with the DNMT inhibitor 5-Aza-2′-deoxycytidine (5-Aza-CdR) induces CpG island hypomethylation and facilitates MMP-9 transcription. This increase in MMP-9 expression results in a significant enhancement of H3NT proteolysis and OCP cell differentiation. On the other hand, despite an increase in levels of H3K18ac, treatment with the HDAC inhibitor trichostatin A (TSA) leads to impairment of osteoclastogenic gene expression. Mechanistically, TSA treatment of OCP-induced cells stimulates H3K27ac with accompanying reduction in H3K27me1, which is a key modification to facilitate stable interaction of MMP-9 with nucleosomes for H3NT proteolysis. Moreover, hypomethylated osteoclastogenic genes in 5-Aza-CdR-treated cells remain transcriptionally inactive after TSA treatment, because H3K27 is highly acetylated and cannot be modified by G9a.

**Conclusions:**

These findings clearly indicate that DNA methylation and histone modification are important mechanisms in regulating osteoclastogenic gene expression and that their inhibitors can be used as potential therapeutic tools for treating bone disorders.

**Electronic supplementary material:**

The online version of this article (10.1186/s13072-019-0270-0) contains supplementary material, which is available to authorized users.

## Background

Bone is a dynamic tissue that undergoes continuous remodeling to control skeletal regeneration and development [1, 2]. This remodeling process is highly orchestrated and tightly regulated by bone-forming osteoblasts and bone-resorbing osteoclasts [3–7]. Osteoclasts are large multinucleated cells that differentiate from the fusion of mononuclear hematopoietic precursors upon stimulation by macrophage colony-stimulating factor (M-CSF) and receptor activator of nuclear factor kappa B ligand (RANKL) [8, 9]. When RANKL binds to its cognate receptor RANK on osteoclast precursor (OCP) cell membrane, multiple signaling pathways are activated to induce the expression of key osteoclastogenic factors [1, 10]. Elevated levels of these factors in OCP-induced cells trigger the expression of downstream genes for the initiation and tight regulation of osteoclast differentiation and function [1, 10]. An abnormal increase in osteoclast formation and activity is a major pathological factor and leads to some bone diseases such as osteoporosis, where resorption exceeds formation causing decreased bone density and increased bone fractures [11, 12]. Therefore, identification and characterization of osteoclastogenic factors have a great therapeutic value for the treatment of bone disorders caused by excessive osteoclastic bone resorption.

Although a number of factors have been reported to be expressed in OCP-induced cells, how the timing and levels of their expression are regulated during the process of differentiation is not well understood. Nevertheless, it has become clear that epigenetic alterations are important in controlling osteoclastogenic gene transcription as well as driving osteoclast formation and activity [13, 14]. These epigenetic changes include DNA methylation at CpG dinucleotides and posttranslational modifications of histone tails [15–20]. Adding additional levels of complexity to these epigenetic processes, our recent study identified an unexpected role for matrix metalloproteinase 9 (MMP-9) in catalyzing H3 N-terminal tail (H3NT) proteolysis and conferring active expression properties to osteoclast-specific genes during osteoclast differentiation [21]. In this report, we demonstrated that the nuclear localization and H3NT cleaving activity of MMP-9 are necessary for transcription of osteoclastogenic genes in OCP-induced cells. Since abrogation of H3NT proteolysis by MMP-9 shRNA and inhibitors resulted in defective osteoclastogenesis under RANKL treatment conditions, the role for MMP-9-dependent H3NT proteolysis in osteoclast differentiation was also firmly established [21]. Beyond these initial works, our follow-up experiments showed that MMP-9 enzymatic activity toward nucleosomal H3NTs is dependent on p300-mediated H3K18ac and G9a-mediated H3K27me1, thus establishing important osteoclastogenic functions for p300 and G9a [22].

As an epigenetic signaling tool, DNA methylation is associated with transcriptional repression by either hindering transcription factor binding or blocking active histone modifications to induce chromatin condensation [17, 23–29]. Therefore, in addition to H3NT proteolysis and histone modification, DNA methylation could be another process through which osteoclast formation and differentiation are regulated. Consistent with this view, a recent report indicated that DNA methyltransferase (DNMT) 3A, one of the de novo DNMTs, exerts its influence on the differentiation capacity of OCP cells by altering the expression of IRF8 [30]. It also has been proposed that DNA methylation changes are involved in the differentiation dynamics of OCP-induced cells and stabilization of osteoclast phenotypes [31]. Although these results support the functional importance of DNA methylation in the regulation of osteoclast differentiation, whether the observed effects of DNA methylation occur through inactivation of MMP-9-dependent H3NT proteolysis and osteoclastogenic gene expression has not yet been investigated.

Given that RANKL-induced formation of mature osteoclasts is epigenetically regulated, we hypothesized that DNMT and histone deacetylase (HDAC) inhibitors can generate distinct effects on MMP-9-dependent H3NT proteolysis and osteoclastogenic gene expression. In this study, we attempted to explore this possibility using isolated murine bone marrow macrophages as OCP cells. We demonstrated that the DNMT inhibitor 5-Aza-CdR significantly facilitated RANKL-induced OCP cell differentiation through CpG demethylation and transcriptional activation of MMP-9 gene. Contrarily, however, treatment of OCP cells with the HDAC inhibitor TSA suppressed osteoclast differentiation. The observed effects were the result of TSA-mediated stimulation of H3K27ac that led to a decrease in H3K27me1 and thus MMP-9 localization at target genes. Moreover, when OCP cells were treated with the combination of 5-Aza-CdR and TSA, TSA was found to exert dominant effects over 5-Aza-CdR.

## Results

### 5-Aza-CdR stimulates MMP-9 expression and osteoclastogenesis

**Fig. 1:**
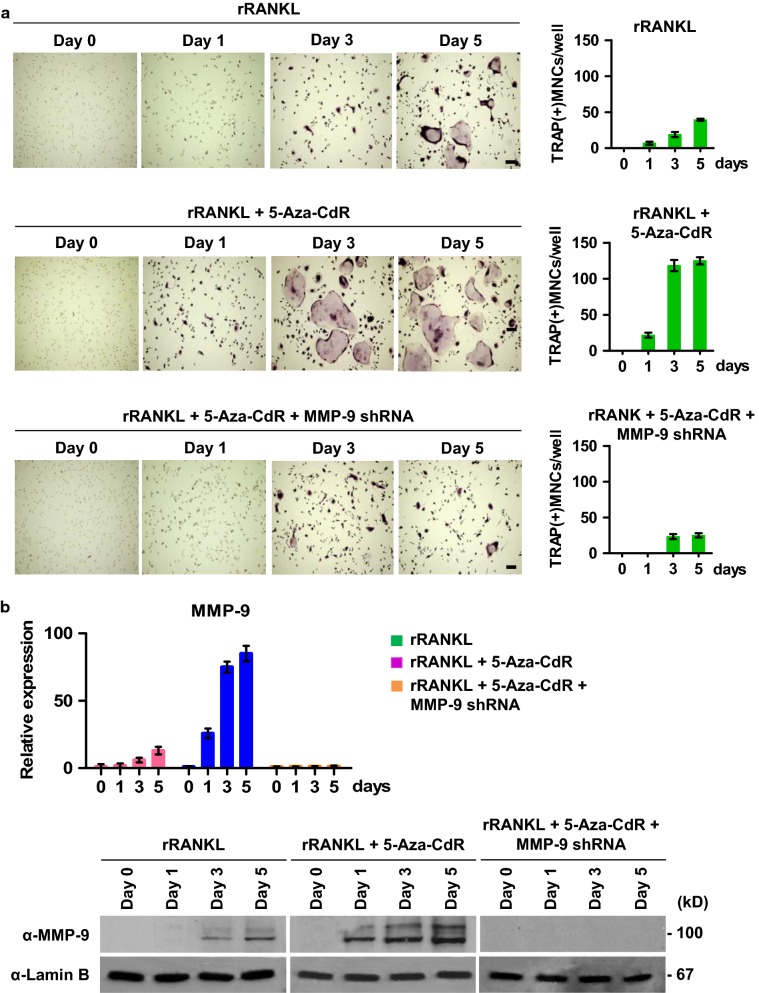
Effects of 5-Aza-CdR treatment on osteoclastogenesis and MMP-9 expression. **a** Mock-depleted (upper and middle panels) or MMP-9-depleted (lower panel) OCP cells were incubated with RANKL in the absence (upper panel) or presence (middle and lower panels) of 5-Aza-dC for 0, 1, 3, or 5 days. Cells were fixed with formaldehyde, stained for TRAP and photographed under a light microscope (10 ×) (left). Representative images of osteoclasts are shown (Scale bar, 100 µm). TRAP-positive multinucleated cells [TRAP(+)MNCs] containing three or more nuclei and a full actin ring were counted as osteoclasts. **b** After treating OCP-induced cells as in **a**, the levels of MMP-9 expression were measured by qRT-PCR (upper panel) with primers in Methods section and Western blotting (lower panel) with MMP-9 antibody. Lamin B antibody was used as a loading control

Because DNA methylation has been implicated in regulatory networks controlling osteoclast differentiation [30–33], we decided to examine the extent to which DNMT inhibitors affect differentiation capacity of OCP cells. In our assay system, OCP cells were prepared from adult mouse long bone and synchronously differentiated into mature osteoclasts in α-minimal essential medium (α-MEM) supplemented with soluble recombinant RANKL for 0, 1, 3 and 5 days. In this setting, mononuclear OCP cells start fusing together to form multinuclear tartrate-resistant acid phosphatase (TRAP)^+^ osteoclasts after 3 days of RANKL treatment (Fig. 1a, upper panel). When OCP-induced cells were exposed to five different concentrations (1, 3, 5, 10 and 20 μM) of the DNMT inhibitor 5-Aza-CdR, we found that 5-Aza-CdR significantly stimulated RANKL-induced osteoclastogenesis at a final concentration of 5 μM (Additional file 1: Fig. S1a). In contrast, only a modest enhancement of OCP cell differentiation was observed after treatment with 1, 3, 10 or 20 μM 5-Aza-CdR. Moreover, the fact that 5-Aza-CdR had no effects on osteoclast viability at 5 μM concentration indicates that 5-Aza-CdR directly modulates the differentiation potential of OCP cells (Fig. 1a, middle panel and Additional file 1: Fig. S1b). Additionally, 5-Aza-CdR treatment of MMP-9-depleted OCP-induced cells was unable to augment differentiation capacity, results indicative of the requirement of MMP-9 for the observed action of 5-Aza-CdR (Fig. 1a, lower panel).

Considering that MMP-9 plays an essential role in osteoclastogenesis [21, 22, 34], we next investigated the impact of 5-Aza-CdR treatment on MMP-9 expression. When total RNA was isolated and analyzed by RT-qPCR, we observed that treating OCP-induced cells with 5-Aza-CdR enhanced MMP-9 expression to levels 10–25-fold higher than OCP-induced cells treated with vehicle control at days 1, 3 and 5 of differentiation (Fig. 1b, upper panel). It was also apparent in our Western blot analysis of nuclear fractions from OCP-induced cells that MMP-9 protein levels increased in parallel with RNA levels after 5-Aza-CdR treatment (Fig. 1b, lower panel). In these Western blottings, we detected an additional slowly migrating minor band. This higher molecular weight band represents the uncleaved pro-MMP-9, and the nuclear level of this inactive form was also increased following 5-Aza-CdR treatment. In the same experiment, Lamin B expression levels were unchanged. Therefore, the increase in MMP-9 expression observed following 5-Aza-CdR treatments is specific. These and the above-noted results underscore the notion that 5-Aza-CdR treatment can up-regulate MMP-9 expression and augment osteoclastogenic potential of OCP-induced cells.

### 5-Aza-CdR decreases DNA methylation at the MMP-9 locus and facilitates H3NT proteolysis

**Fig. 2:**
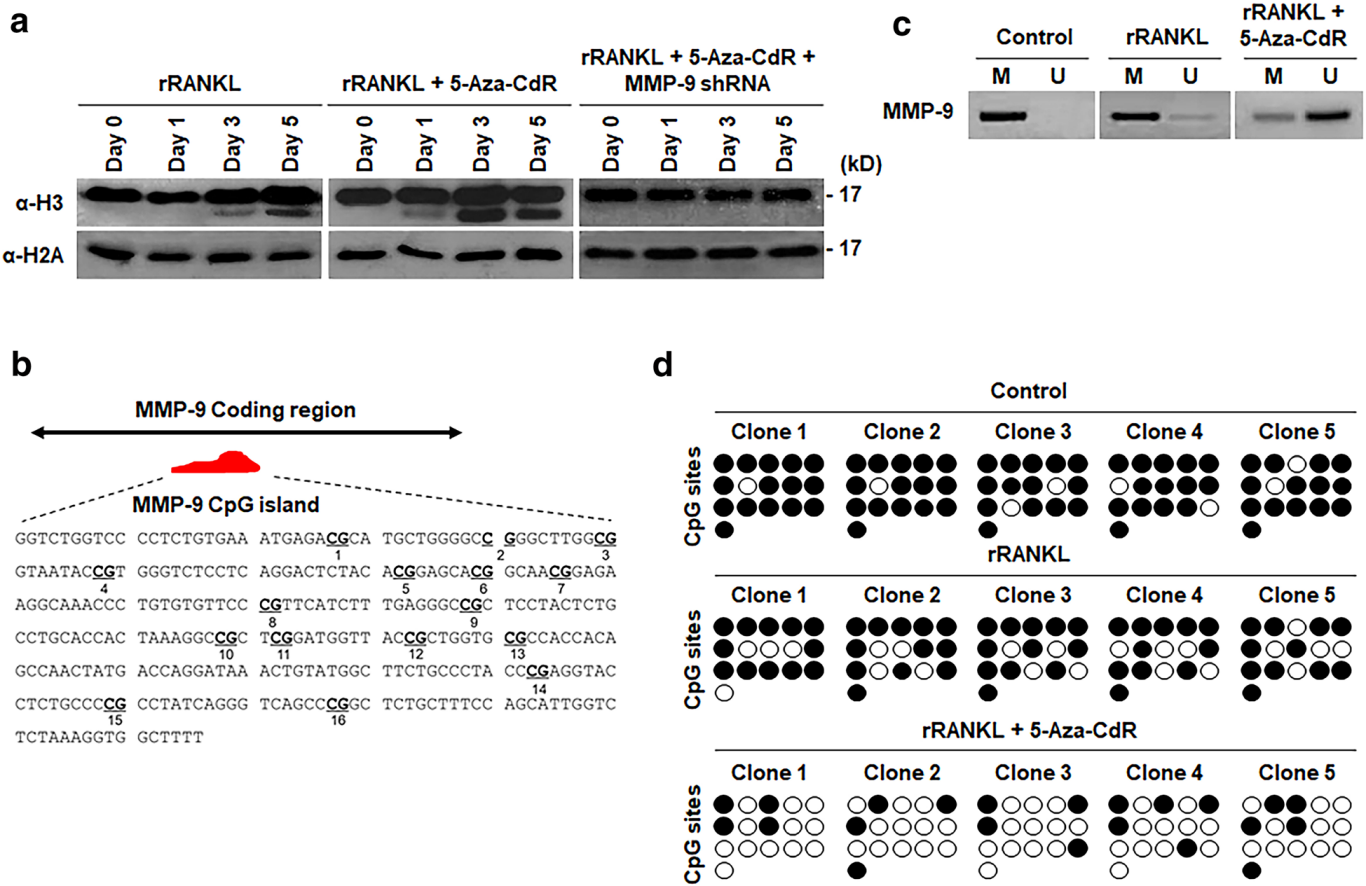
Effects of 5-Aza-CdR treatment on MMP-9 CpG methylation and H3NT proteolysis. **a** Mock-depleted or MMP-9-depleted OCP-induced cells were cultured with or without 5-Aza-dC for 0, 1, 3 and 5 days. Chromatin was extracted, and the extent of H3NT proteolysis was analyzed by Western blotting with H3 C-terminal tail antibody. H2A was used as a loading control. **b** The sequence of the MMP-9 CpG island locus (NCBI accession: NC_000068, region: 164950229 to 164950544) containing the 16 CpGs that are written in bold and underlined. **c** Genomic DNA was obtained from OCP-induced cells that were grown in the presence or absence of 5-Aza-CdR for 5 days. The purified DNA molecules were subjected to sodium bisulfite conversion reaction and then analyzed by methylation-specific PCR of the MMP-9 CpG island. M, methylated; U, unmethylated. **d** Bisulfite sequencing analysis of the MMP-9 CpG island locus using genomic DNA prepared as in **c**. White circles, unmethylated CpGs; black circles, methylated CpGs

Our recent investigation established MMP-9 as a novel H3NT protease required for H3NT proteolysis observed during the process of osteoclast formation [21, 22]. Since 5-Aza-CdR treatment of OCP-induced cells led to a sharp increase in MMP-9 expression (Fig. 1b), we wondered whether H3NT proteolysis is also affected by 5-Aza-CdR treatment. In order to check this possibility, we extracted chromatin from control and OCP-induced cells after 0, 1, 3 or 5 days of 5-Aza-CdR treatment and analyzed by Western blotting with H3 C-terminal antibody. In agreement with our published data, we detected a fast migrating H3 band representing H3NT proteolysis during osteoclast differentiation (Fig. 2a, left lane). In parallel experiments in which H3NT proteolysis was analyzed in 5-Aza-CdR-treated OCP cells over the same time period, much higher levels of H3NT proteolysis were observed (Fig. 2a, middle lane). On the contrary, however, 5-Aza-CdR treatment was no longer capable of positively regulating H3NT proteolysis, when MMP-9 was depleted in OCP-induced cells (Fig. 2a, right lane). These data are supportive of the idea that stimulatory effects of 5-Aza-CdR on osteoclast differentiation pathway are dependent on MMP-9-dependent H3NT proteolysis.

Given the demonstrated effects of 5-Aza-CdR treatment on MMP-9 expression and H3NT proteolysis, we also wanted to study how 5-Aza-CdR treatment affects DNA methylation levels of MMP-9 gene. Toward this end, we analyzed the DNA sequence of the MMP-9 gene using a CpG island search program and identified a 316-bp-long CpG island that starts at 164950229 and extends up to 164950544 in the coding region of the gene (Fig. 2b). To examine changes in DNA methylation status in this CpG island, we first conducted methylation-specific PCR with DNA isolated from mock-treated or 5-Aza-CdR-treated OCP-induced cells after converting all unmethylated cytosines to uracil. As shown in Fig. 2c, 5-Aza-CdR treatment decreased the band intensity of the methylated CpG island and increased the band intensity of the unmethylated CpG island. To further confirm the demethylating effects of 5-Aza-CdR on the MMP-9 coding region in OCP-induced cells, DNA bisulfite sequencing was performed. Primers were designed to amplify 16 different fragments that are located within the CpG island of the MMP-9 coding region (Fig. 2b). PCR products were then subcloned into a pGEM-T Easy vector, and 5 separate subclones were sequenced. In uninduced control cells, 16 CpG sites in the MMP-9 coding region were highly methylated, with overall methylation levels of ~ 90% (Fig. 2d, upper level). Approximately 30% reduction in methylation levels of the CpG sites was detected after RANKL-induced differentiation of OCP cells (Fig. 2d, middle level). Meanwhile, in a parallel experiment in which OCP-induced cells were treated with 5-Aza-CdR, CpG methylation levels in the MMP-9 coding region were decreased to nearly 25% (Fig. 2d, lower level), clearly indicating that 5-Aza-CdR is able to induce demethylation of the MMP-9 coding region in OCP-induced cells.

### 5-Aza-CdR promotes MMP-9 function at target genes

**Fig. 3:**
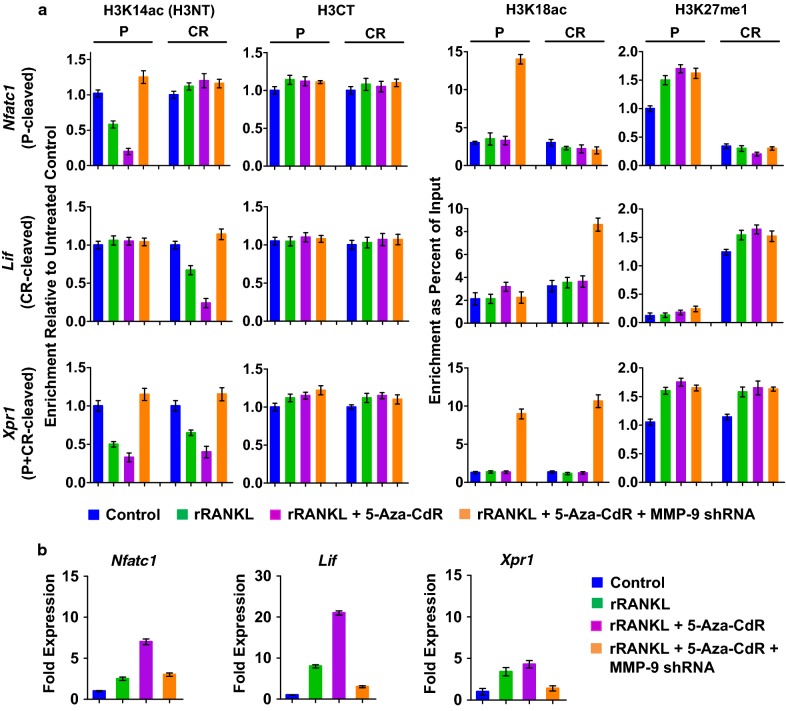
Effects of 5-Aza-CdR treatment on MMP-9 transactivation function. **a** Mock-depleted or MMP-9-depleted OCP-induced cells were cultured in the presence or absence of 5-Aza-CdR for 5 days and subjected to ChIPac/ChIP analysis using the antibodies indicated on the top. Precipitation efficiencies were determined for the promoter (P) and coding region (CR) of Nfatc1 (P-cleaved), Lif (CR-cleaved) and Xpr1 (P + CR-cleaved) genes by qPCR with primers used in our previous study [21, 22] and Methods section. **b** RT-qPCR assays were performed to determine fold changes in Nfatc1, Lif and Xpr1 expression in mock-depleted or MMP-9-depleted OCP-induced cells after 5 days of culture in the presence or absence of 5-Aza-CdR

Having established the stimulatory effects of 5-Aza-CdR treatment on MMP-9 expression and osteoclastogenesis, we next sought to explore whether 5-Aza-CdR treatment also influences H3NT proteolytic reactions at MMP-9 target genes. We recently developed a technique called ChIP of acetylated chromatin (ChIPac) to determine the genomic sites targeted for H3NT proteolysis during osteoclastogenesis [21]. This new method utilizes methylene blue to cross-link chromatin and acetic anhydride to acetylate all lysine residues in fragmented chromatin. Intact H3NT-containing chromatin was then precipitated with anti-H3K14ac antibody and subjected to quantitative PCR or next-generation sequencing analysis. A reduced product in PCR or sequencing reactions relative to the control reactions using chromatin precipitated with an H3CT antibody is indicative of H3NT proteolysis. During osteoclast differentiation, MMP-9 mediates H3NT cleavage in promoter, coding region or both for target gene transcription in a gene-specific manner. Thus, we examined the levels of H3NT proteolysis at Nfatc1, Lif and Xpr1 genes representing each H3NT-cleaved group in mock-treated or 5-Aza-CdR-treated OCP-induced cells by ChIPac-qPCR. In agreement with our published data [21, 22], RANKL treatment of OCP cells resulted in a rapid cleavage of H3NT in the P, CR, and both regions of Nfatc1, Lif and Xpr1 genes, respectively (Fig. 3a). Treatment of OCP-induced cells with 5-Aza-CdR led to markedly higher levels of H3NT proteolysis at target genes relative to mock-treated cells (Fig. 3a). These results were further corroborated by the knockdown experiments in which shRNA-mediated depletion of MMP-9 almost completely crippled such elevated H3NT proteolysis in 5-Aza-CdR-treated OCP-induced cells. Also, our examination of the levels of H3K18ac and H3K27me1 confirmed that 5-Aza-CdR treatment did not have any significant effects on these modifications (Fig. 3a). Thus, the observed augmentation of H3NT proteolysis could not be explained by a possible impact of 5-Aza-CdR treatment on p300 and G9a expression and their enzymatic activities. For the purpose of examining whether 5-Aza-CdR treatment also influences MMP-9 transactivation during osteoclastogenesis, we exposed OCP cells to 5-Aza-CdR and measured the levels of transcriptional activation of MMP-9 target genes. Congruent to previously published data [21, 22], Nfatc1, Lif and Xpr1 genes were effectively activated upon RANKL-induced osteoclast differentiation (Fig. 3b). When OCP-induced cells were treated with 5-Aza-CdR, much higher levels of Nfatc1, Lif and Xpr1 gene transcription were evident (Fig. 3b). Since 5-Aza-CdR treatment was unable to enhance target gene expression upon depletion of MMP-9 by RNAi (Fig. 3b), MMP-9 was a critical target of the observed effects of 5-Aza-CdR treatment. Overall, these observations suggest that 5-Aza-CdR treatment has positive impact on accurate localization and transactive function of MMP-9 at pro-osteoclastogenic genes.

### TSA inhibits osteoclast differentiation and H3NT proteolysis

**Fig. 4:**
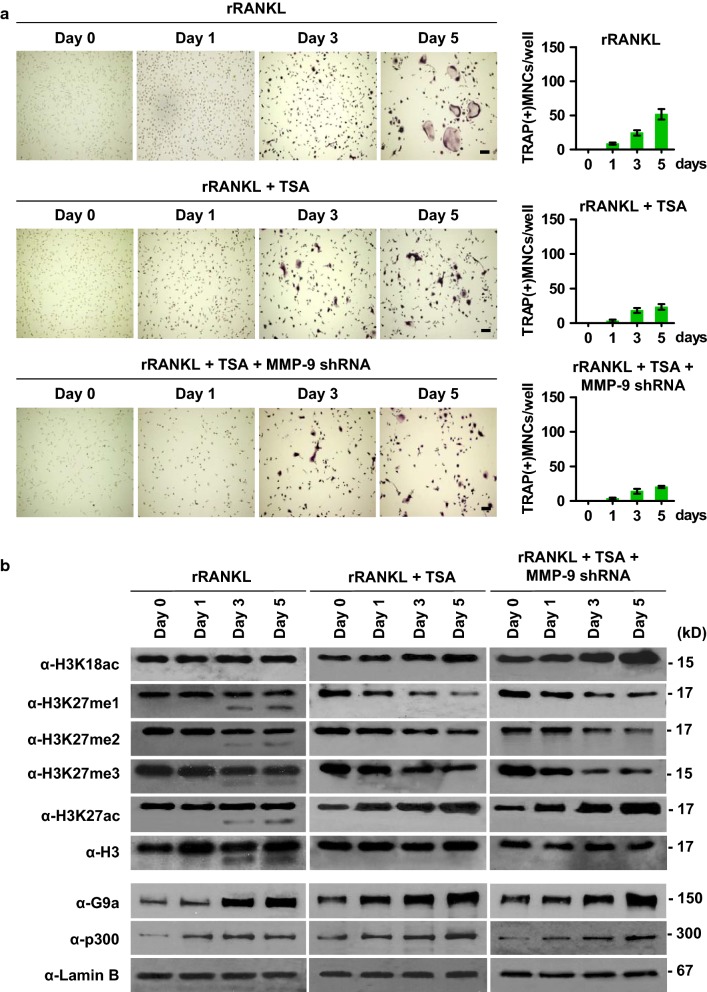
Effects of TSA treatment on osteoclastogenesis and H3K27me1. **a** Mock-depleted or MMP-9-depleted OCP-induced cells were grown with or without TSA for 0, 1, 3 and 5 days and analyzed by TRAP staining (left panel) and total counting (right panel). **b** Mock-depleted or MMP-9-depleted OCP-induced cells were cultured as in (**a**), and chromatin was extracted and analyzed by Western blotting with the antibodies indicated on the left

We next wanted to test the possibility that the HDAC inhibitor TSA can influence RANKL-induced H3NT proteolysis and OCP cell differentiation. Given the demonstrated reliance of MMP-9 on H3K18ac for its enzymatic activity toward nucleosomal H3NT [21], it was reasonable to expect that TSA treatment will stimulate H3K18ac and thus osteoclastogenesis. Surprisingly, however, our differentiation assays led us to discover that treatment of OCP cells with 20 nM TSA produces a significant inhibition in the differentiation capacity of OCP-induced cells (Fig. 4a, middle panel). In parallel assays, knockdown of MMP-9 failed to generate more pronounced effects in TSA-treated OCP-induced cell cultures (Fig. 4a, lower panel), indicating that TSA exerts its inhibitory effects on osteoclastogenesis in a MMP-9-dependent manner. Although TSA at concentrations higher than 20 nM was somewhat more inhibitory, the observed inhibition was likely due to a negative effect on the growth or viability, rather than the differentiation, of OCP cells (Additional file 2: Fig. S2). For this reason, we conducted all of the aforementioned assays with 20 nM TSA.

Since p300-mediated H3K18ac and G9a-mediated H3K27me1 are essential for MMP-9-dependent H3NT proteolysis [21, 22], we postulated that the observed inhibition of osteoclastogenesis after TSA treatment is generated by changes in these modification processes. To check this possibility, soluble chromatin was prepared from the nuclei of control and OCP-induced cells after TSA treatment for 0, 1, 3 or 5 days and subjected to Western blot analysis. As shown in Fig. 4b, higher levels of H3K18ac were retained in chromatin purified from TSA-treated OCP-induced cells than mock-treated control cells. Contrarily, TSA treatment of OCP-induced cells resulted in a substantial decrease in the levels of H3K27me, especially H3K27me1 and H3K27me3. The marked reduction in H3K27me1 caused by TSA treatment was surprising, because increased G9a expression was apparent from our Western blot analysis of TSA-treated, OCP-induced cells (Fig. 4b). Given that H3K27 can be both methylated and acetylated, a possible explanation for this observation is that H3K27ac becomes the dominant modification and blocks G9a-mediated H3K27me1 after TSA treatment. Consistent with this idea, we detected the hyperacetylation of H3K27 in chromatin samples from TSA-treated, OCP-induced cells (Fig. 4b). In the same experimental condition, TSA treatment did not appreciably alter p300 expression levels (Fig. 4b), thus implying that p300 is dispensable for the H3K27ac reaction. p300-independency of the H3K27ac reaction was further supported by knockdown experiments, demonstrating that H3K27ac was not affected by p300 depletion in TSA-treated, OCP-induced cells (Additional file 3: Fig. S3).

**Fig. 5:**
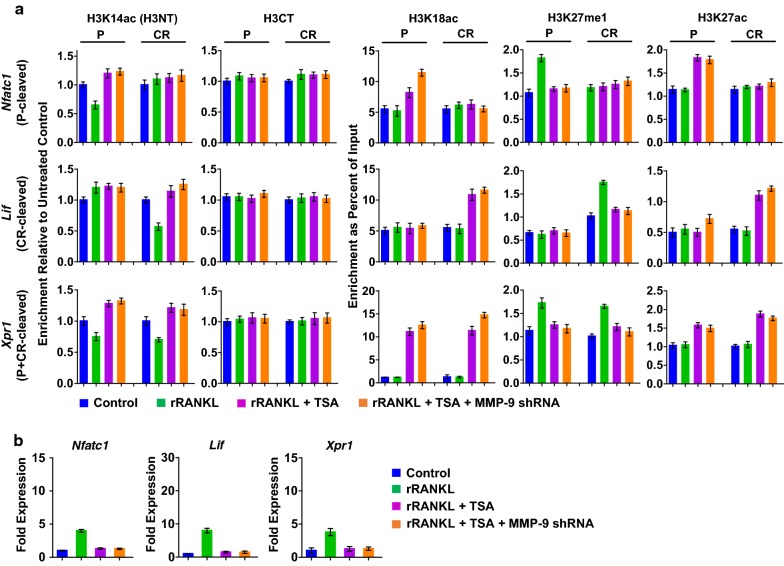
Effects of TSA treatment on MMP-9 transactivation function. **a** Mock-depleted or MMP-9-depleted OCP-induced cells were cultured in the presence or absence of TSA for 5 days and analyzed by ChIPac/ChIP-qPCR. **b** Mock-depleted or MMP-9-depleted OCP-induced cells were treated with TSA as in **a**, and relative expression levels of Nfatc1, Lif and Xpr1 genes were determined by qRT-PCR

In order to determine whether TSA influences similarly on MMP-9 transactivation pathway, ChIPac/ChIP-qPCR assays were carried out, using chromatin prepared from mock-treated or TSA-treated OCP-induced cells. Figure 5a shows that H3NT proteolysis was enriched at the promoter and/or coding region of Nfatc1, Lif and Xpr1 genes in mock-treated OCP-induced cells but largely disappeared upon TSA treatment. The target genes harbor high levels of H3K27ac and lose H3K27me1 after 5-day TSA treatment, again suggesting monomethylation to acetylation switch at H3K27. Essentially, identical results were obtained when MMP-9-depleted OCP-induced cells were treated with TSA. The inhibitory effects of TSA treatment on osteoclastogenesis were also verified by monitoring changes in the expression of NFATc1, Lif and Xpr genes in mock-treated and TSA-treated OCP-induced cells. The observed repression appears to be independent of cellular MMP-9 levels, since depletion of MMP-9 did not show a corresponding effect on the expression of the target genes tested in TSA-treated OCP-induced cells (Fig. 5b). Collectively, these results indicate that TSA impairs MMP-9-dependent H3NT proteolysis and osteoclastogenesis by targeting H3K27me1-dependent function of MMP-9.

### TSA exerts dominant effects in OCP-induced cells co-treated with TSA and 5-Aza-CdR

**Fig. 6:**
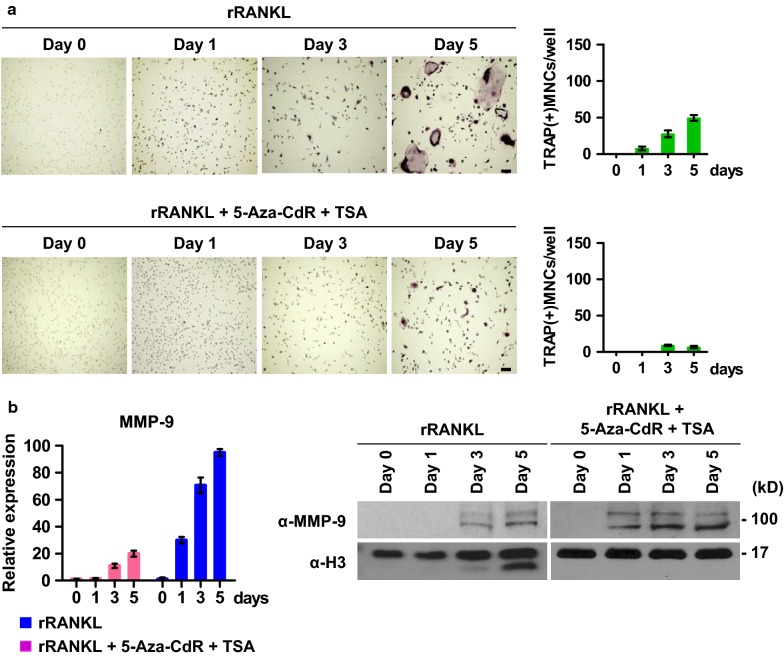
Effects of TSA and 5-Aza-CdR co-treatment on osteoclastogenesis. **a** OCP-induced cells were treated with the combination of 5-Aza-CdR and TSA (lower panel) or DMSO control (upper panel) for 0, 1, 3 and 5 days and stained for TRAP. **b** OCP-induced cells were treated as in **a**, and changes in MMP-9 expression were analyzed by RT-qPCR (left panel) and Western blot (right panel). Chromatin was also isolated and analyzed by Western blotting to assess the effects of the combined treatment on H3NT proteolysis (right panel)

**Fig. 7:**
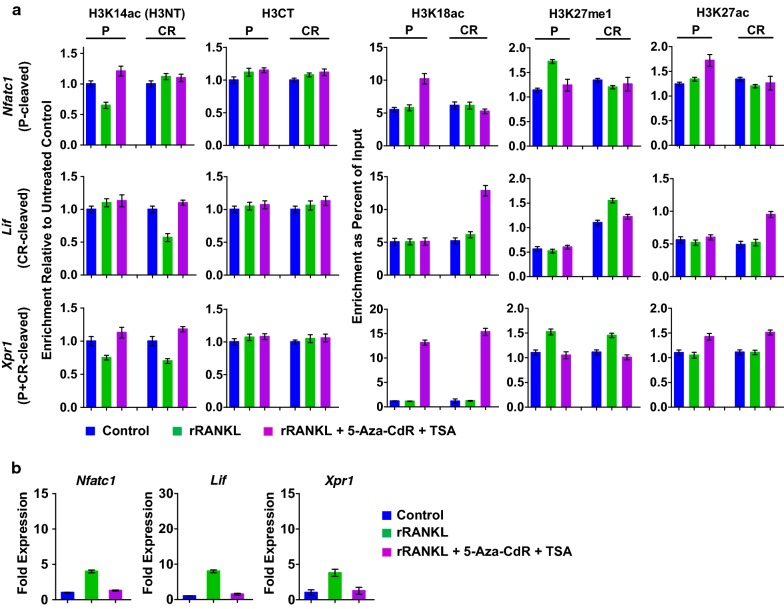
Effects of TSA and 5-Aza-CdR co-treatment on MMP-9 transactivation function. After treating with the combination of 5-Aza-dC and TSA for 5 days, OCP-induced cells were subjected to ChIPac/ChIP-qPCR (**a**) to measure H3NT proteolysis levels and RT-qPCR (**b**) to quantitate Nfatc1, Lif and Xpr1 expression levels

As an extension of the above-described studies that established opposite effects of 5-Aza-CdR and TSA treatments on MMP-9-dependent H3NT proteolysis and osteoclastogenesis, it was also important to evaluate the effects of combined treatment with 5-Aza-CdR and TSA inhibitors. For this objective, we treated OCP-induced cells with 5 μM 5-Aza-CdR and 20 nM TSA, which are the concentrations exhibiting minimal effects on OCP cell viability (Additional files 1 and 2: Fig. S1b and S2b). Five days after treatment, OCP-induced cells were subjected to TRAP assays to analyze changes in their differentiation potential (Additional file 2: Fig. S2a). Our assays revealed that combined treatment with the DNMT inhibitor 5-Aza-CdR and HDAC inhibitor TSA significantly inhibited the formation of mature, multinucleated osteoclasts from OCP-induced cells (Fig. 6a). These effects were paralleled by an increase in the levels of MMP-9 expression (Fig. 6b, left panel). Remarkably, however, H3NT proteolysis was completely abolished following a co-treatment with 5-Aza-CdR and TSA, as evidenced by the absence of a faster migrating H3 band in Western blotting (Fig. 6b, right panel). Moreover, and as shown in Fig. 7a, ChIPac and ChIP assays in 5-Aza-CdR/TSA-co-treated OCP-induced cells showed that NFATc1, Lif and Xpr1 genes harbor high levels of H3K18ac, H3K27me1 and cleaved H3 protein in the P and/or CR regions. The observed enrichment of H3K27me1 and H3NT cleavage largely disappeared after combined treatment with 5-Aza-CdR and TSA. Since an increase in H3K27ac was observed following 5-Aza-CdR and TSA treatment, these results support the dominant action of TSA over 5-Aza-CdR in regulating MMP-9 target gene pathways necessary for proficient osteoclast differentiation. In fact, consistent with this concept, our RT-qPCR analyses demonstrated that 5-Aza-CdR and TSA treatments of OCP-induced cells caused two- to fivefold decreases in the expression of NFATc1, Lif and Xpr1 genes (Fig. 7b).

## Discussion

Our recent investigation revealed that MMP-9 is the protease responsible for catalyzing H3NT proteolysis and activating genes encoding master regulators of osteoclastogenesis in response to RANKL treatment [21]. We also established H3K18ac and H3K27me1 as prerequisite marks for the recruitment and enzymatic activity of MMP-9 toward H3NT at target genes [21, 22]. However, nothing is known about the influence of DNA methylation on MMP-9-dependent H3NT proteolysis and gene activation reactions in OCP-induced cells. Since a large body of evidence links DNA methylation to gene silencing [17, 24, 35, 36], we hypothesized that DNA methylation attenuates H3NT proteolysis and that such an effect is associated with inhibition of osteoclast differentiation. The current study supports this hypothesis by showing that treatment of OCP-induced cells with the DNMT inhibitor 5-Aza-CdR stimulates MMP-9-dependent H3NT proteolysis and augments the expression of a group of genes, whose products are critical for the formation of multinucleated osteoclasts and their bone-resorbing activities. Mechanistically, exposure of OCP-induced cells to 5-Aza-CdR enhances MMP-9 expression and H3NT cleavage under RANKL-activated differentiation conditions. Also consistent with its previously demonstrated function in osteoclastogenesis [21, 22], MMP-9-dependent H3NT proteolysis in 5-Aza-CdR-treated OCP-induced cells significantly increased the number of differentiated osteoclasts and activated transcription of osteoclastogenic genes. To the best of our knowledge, this is the first report to demonstrate that DNA methylation is one of the mechanisms modulating MMP-9 expression during osteoclast differentiation and that 5-Aza-CdR-induced MMP-9 production is associated with the activation of osteoclastogenic transcription pathways.

Since MMP-9 requires H3K18ac for its enzymatic activity toward H3NT [21, 22], HDAC inhibitors were also thought to be effective in blocking H3NT proteolysis and osteoclastogenesis. With this expectation, the HDAC inhibitor TSA has also been tried in our assays. It was, however, somewhat surprising to see that the addition of TSA to OCP-induced cell culture does not stimulate, but rather inhibits MMP-9-dependent H3NT proteolysis in OCP-induced cells. In addition, TSA treatment substantially increased the levels of H3K27ac, correlated with low levels of H3K27me1 and osteoclast differentiation. These results strongly argue that TSA targets HDAC activities removing acetyl groups from H3K27ac to sequester G9a-mediated H3K27me1. In an extension of this initial discovery, we showed further that the presence of TSA in OCP cell differentiation reactions contributes to the inhibition of MMP-9-dependent H3NT proteolysis through mechanisms involving disruption of H3K27me1-MMP-9 interaction, leading to abrogation of MMP-9 protease activity targeting H3NTs. This observation highlights the importance of H3K27me1 in the regulation of MMP-9-dependent H3NT proteolysis and confirms that accurate recruitment of MMP-9 to target genes is vital for the control of H3NT proteolysis and RANKL-induced osteoclastogenesis. Our findings also suggest that HDAC activity is a promising target for therapeutic interventions that involve modulating osteoclastic bone resorption, as HDAC inhibition by TSA will decrease the differentiation rate of OCP cells.

Because TSA treatment switched H3K27 modification status from monomethylation to acetylation and thus interfered with the binding of MMP-9 to target nucleosomes, we expected Aza treatment to be ineffective when used together with TSA. Indeed, although 5-Aza-CdR alone increased the differentiation capacity of RANKL-treated OCP cells, the combination of 5-Aza-CdR with TSA generated a significant impairment of MMP-9-dependent transactivation and osteoclast differentiation. The observed inhibition coincides with a stable reduction in H3K27me1 and an apparent decrease in H3NT proteolysis, again underscoring the importance of G9a-mediated H3K27me1 as an early step necessary for RANKL-induced MMP-9-dependent osteoclastogenesis. Although we have mainly focused on 5-Aza-CdR and TSA, the present study could serve as an important starting point for a broader characterization of properties shared by other DNMT and HDAC inhibitors. It is tempting to speculate that exposure of OCP-induced cells to other DNMT and HDAC inhibitors might lead to similar outcomes, via affecting MMP-9 activity toward H3NTs. Also of note, our recent analyses revealed that cancer cells residing in bone express and secrete a group of factors that stimulate differentiation and maturation of OCP-induced cells [37, 38]. The unbalanced generation of osteoclasts leads to a massive bone resorption and releases the growth factors stored in the bone matrix, promoting metastatic tumor growth. This feed-forward vicious cycle creates a fertile microenvironment for cancer cell growth in bone to drive the devastating effects of bone destruction. DNA methylation and histone acetylation are also known to function in these bone metastatic cancer cells [37–39]. Thus, a combination of 5-Aza-CdR and TSA that target both OCP-induced cells and bone-residing metastatic cancer cells may also have great therapeutic value in treating osteoporosis and other bone-erosive diseases such as rheumatoid arthritis and metastasis associated with bone loss.

## Conclusions

Our study demonstrates that 5-Aza-CdR treatment stimulates MMP-9 protease activity targeting H3NTs and gene transcription by elevating MMP-9 expression levels in OCP-induced cells. On the other hand, TSA treatment interferes with MMP-9-dependent H3NT proteolysis because it changes H3K27me1 to H3K27ac and sequesters MMP-9 recruitment to target genes. These results suggest that 5-Aza-CdR and TSA could be used to establish distinct epigenetic states to regulate localization and function of MMP-9 at pro-osteoclastogenic genes.

## Methods

### OCP cell preparation and differentiation assay

Bone marrow cells were harvested from tibias and femurs of 6- to 8-week-old C57BL/6 mice as recently described [21, 22]. The cells were cultured in α-minimum essential medium (α-MEM) supplemented with 10% fetal bovine serum (FBS) and M-CSF (5 ng/ml) for 12 h. Non-adherent cells were harvested and incubated with M-CSF (30 ng/ml) for 48 h. Old media were replaced with fresh media containing M-CSF and further cultured for 24 h. Floating cells were removed, and adherent cells were used as osteoclast precursor (OCP) cells. For differentiation assays, OCP cells were grown in α-MEM culture medium containing M-CSF (30 ng/ml) and RANKL (5 ng/ml) with or without 5-Aza-CdR or TSA. After being cultured for 0, 1, 3, or 5 days, OCP-induced cells were fixed and stained for tartrate-resistant acid phosphatase (TRAP) using an acid phosphatase leukocyte kit (Sigma) as recently described [21, 22]. TRAP-positive multinuclear cells containing three or more nuclei and a full actin ring were counted as osteoclasts under a light microscope.

### Cell viability assay

OCP cells were seeded in 96-well plates at a density of 0.5 × 10^4^ and treated with 5-Aza-CdR (0–20 µm) or TSA (0-80 nM) for 5 days. Cell viability was assessed by using the MTT (3-(4,5-dimethylthyazol-2-yl)-2,5-diphenyl-tetrazolium bromide) cell growth assay kit (Sigma Aldrich) as detailed previously [40].

### Western blot analysis

Chromatin was isolated from OCP-induced cells after completion of 5-day mock or TSA treatment and analyzed by Western blotting with anti-H3 C-terminal, anti-H3K18ac, anti-H3K27ac, anti-H3K27me1/H3K27me2/H3K27me3 antibodies. Protein samples were also collected from 5-day 5-Aza-CdR-treated OCP-induced cells, and the levels of MMP-9, p300 and G9a were determined by Western blot analysis.

### RNAi, ChIPac/ChIP and RT-qPCR

For the depletion of MMP-9 and p300, OCP cells were transduced with MMP-9 and p300 shRNA and then selected with puromycin (2 μg/ml) as previously described [21, 22]. ChIPac assays were performed using chromatin that was fixed with 10 µM methylene blue and acetylated with 20 mM acetic anhydride as detailed previously [21, 22]. H3K14ac and H3CT antibodies were immobilized on protein A/G-PLUS agarose and used to immunoprecipitate cross-linked chromatin. ChIP assays were conducted with H3K18ac and H3K27me1 antibodies and the ChIP assay kit (Millipore) as recently described [21, 22]. DNA fragments were recovered from precipitated protein–DNA complexes using Qiagen kit after reversal of crosslinking at 65 °C and analyzed by qPCR with the primers that amplify the promoter (P) and coding regions (CR) of Nfatc1 (P-cleaved), Lif (CR-cleaved) and Xpr1 (P + CR-cleaved) genes. For RT-qPCR, total RNA was isolated from OCP-induced cells using the RNeasy kit (Qiagen). After converting RNA to cDNA using iScript cDNA Synthesis Kit (Bio-Rad), RT-PCR was performed using one-step QuantiTect SYBR Green RT-PCR kit (Qiagen) according to the manufacturer’s instructions. The primers used for ChIPac and RT-qPCR are: Nfatc1 (P: 5′-GAAGTGGTAGCCCACGTGAT-3′, 5′-TCTTGGCACCACATAAACCA-3′; CR: 5′-GGGTCAGTGTGACCGAAGAT-3′, 5′-GGAAGTCAGAAGTGGGTGGA-3′; mRNA: 5′-CGTACCTTCCTGCCAATGTT-3′, 5′-TGGTGAGCTGTTGGCTGTAG-3′), Lif (P: 5′-CTCTGGCTGTCCTGGAACTC-3′, 5′-CCAGGACCAGGTGAAACACT-3′; CR: 5′-ATCTTGTGGCTTTGCCAACT-3′, 5′-AGTCCTTGCCTGTCTTTCCA-3′; mRNA: 5′-TACTGCTGCTGGTTCTGCAC-3′, 5′-TGAGCTGTGCCAGTTGATTC-3′), Xpr1 (P: 5′-AGGACCTTCGGAAGAGCAGT-3′, 5′-CAGCAAGCAGCTCATAACCA-3′; CR: 5′-GGTGGGTTCCACTGAAAGAA-3′, 5′-GGTTCCTCTGACCAAAAGCA-3′; mRNA: 5′-CGCAGGTTTGCTACACTTCA-3′, 5′-CCTATGTTGGACACGCTCCT-3′), MMP-9 (mRNA: 5′-CAATCCTTGCAATGTGGATG-3′, 5′-AGTAAGGAAGGGGCCCTGTA-3′), p300 (mRNA: 5′-GAGGAGAGAGGCCCTGAGTT-3′, 5′-CGGTAAAGTGCCTCCAATGT-3′) and G9a (mRNA: 5′-TGCCTATGGTCAGCTCAG-3′, 5′-GGTTCTTGCAGCTTCTCCAG-3′).

### Methylation-specific PCR

Genomic DNA was isolated from mock-treated or 5-Aza-CdR-treated OCP-induced cells using the QIAamp DNA Blood Mini Kit (Qiagen) and subjected to bisulfite conversion using the EpiTect Bisulfite Kit (Qiagen). Methylation-specific PCR was performed with the converted DNA molecules and MMP-9 CpG island-specific primers which were designed by the Methyl Primer Express software. The methylation-specific primer sequences of MMP-9 are: MMP-9 M (5′-TCGTTATGGTTATATTCGGGTC-3′, 5′-AATCGACCCACGTCTAAAAC-3′) and MMP-9 U (5′-GTATTGTTATGGTTATATTTGGGTT-3′, 5′-AATCAACCCACATCTAAAACACC-3′).

### Bisulfite sequencing

Genomic DNA (1 μg) was modified by sodium bisulfite conversion reaction with the EpiTect Bisulfite Kit (Qiagen). The modified DNA was amplified using bisulfite primers, which were specific for the MMP-9 CpG island and designed by the Methyl Primer Express software. After PCR reactions, PCR products were purified with a Gel Extraction Kit (Qiagen) and ligated into the pGEM-T easy vector (Promega). Five separate clones were selected from mock-treated or 5-Aza-CdR-treated OCP cells for bisulfite sequencing analysis. The bisulfite primer sequences are: MMP-9 (5′-GGTTTGGTTTTTTTTGTGAAAT-3′, 5′-AAAAACCACCTTTAAAAACCAA-3′).

### Antibodies

Antibodies were used in this study are as follows: anti-MMP-9, anti-G9a anti-H2A and anti-H3 antibodies from Abcam; anti-H3K14ac and anti-H3K18ac antibodies from Active motif; anti-H3K27ac antibody from Gene Tax; anti-H3K27me1, anti-H3K27me2 and anti-H3K27me3 antibodies from EpiGentek; anti-p300 and anti-Lamin B antibodies from Santa Cruz Biotech.

### Statistical analyses

All quantitative data are presented as mean ± standard deviation (SD). Statistical analyses of datasets were performed with Student’s two-tailed *t* test or two-way ANOVA followed by Bonferroni post hoc test using GraphPad Prism software (GraphPad Software Inc.) which was used for all analyses of the experiments. A *P* value < 0.05 was considered statistically significant.

## Additional files

**Additional file 1.** Effects of increasing concentration of 5-Aza-CdR on OCP cell viability and differentiation. **a** After treating with the indicated concentrations of 5-Aza-CdR for 5 days, OCP-induced cells were stained for TRAP (left) and positive cells were counted (right). **b** OCP cells were treated with 5-Aza-CdR as in (a), and their relative viability was assessed by MTT assay.

**Additional file 2.** Effects of increasing concentration of TSA on OCP cell viability and differentiation. **a** OCP-induced cells were treated with the indicated concentrations of TSA for 5 days and subjected to TRAP staining analysis. **b** OCP cells were treated with TSA as in (a), and their viability was scored by MTT assay.

**Additional file 3.** Analysis of effects of p300 knockdown on H3K27ac in TSA-treated, OCP-induced cells. Mock-depleted or p300-depleted OCP-induced cells were cultured for 0, 1, 3, 5 days in the presence of TSA, and chromatins and nuclear lysates were analyzed by Western blotting with H3K27ac, H3, p300 and Lamin B antibodies.

## Abbreviations

5-Aza-CdR: 5-Aza-2′-deoxycytidine
TSA: trichostatin A
H3NT: histone H3 N-terminal tail
H3K14ac: H3 acetylation at lysine 14
H3K18ac: H3 acetylation at lysine 18
H3K27ac: H3 acetylation at lysine 27
H3K27me1: H3 monomethylation at lysine 27
H3K27me2: H3 dimethylation at lysine 27
H3K27me3: H3 trimethylation at lysine 27
RANK: receptor activator of NF-κB ligand
MMPs: matrix metalloproteinases
OCP: osteoclast precursor
TRAP: tartrate-resistant acid phosphatase
ChIPac: ChIP of acetylated chromatin
