# Supplementary figures and images for: DNMT and HDAC inhibitors modulate MMP-9-dependent H3 N-terminal tail proteolysis and osteoclastogenesis
Source: Epigenetics Chromatin. 2019 Apr 16;12:25. doi: 10.1186/s13072-019-0270-0 (PMC6466663; doi:10.1186/s13072-019-0270-0)

# Additional file 1

a

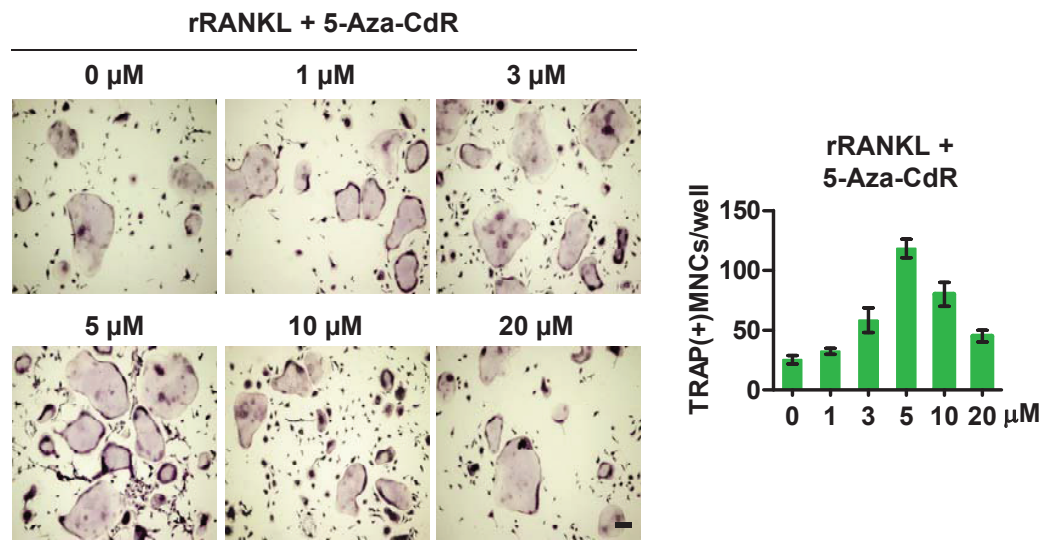

b

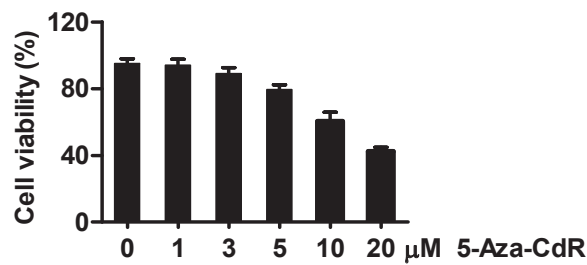

Supplement: Supplementary file 1 — Additional file 1. Effects of increasing concentration of 5-Aza-CdR on OCP cell viability and differentiation. a After treating with the indicated concentrations of 5-Aza-CdR for 5 days, OCP-induced cells were stained for TRAP (left) and positive cells were counted (right). b OCP cells were treated with 5-Aza-CdR as in (a), and their relative viability was assessed by MTT assay. [file 13072_2019_270_MOESM1_ESM.pdf]

Additional file 2

a

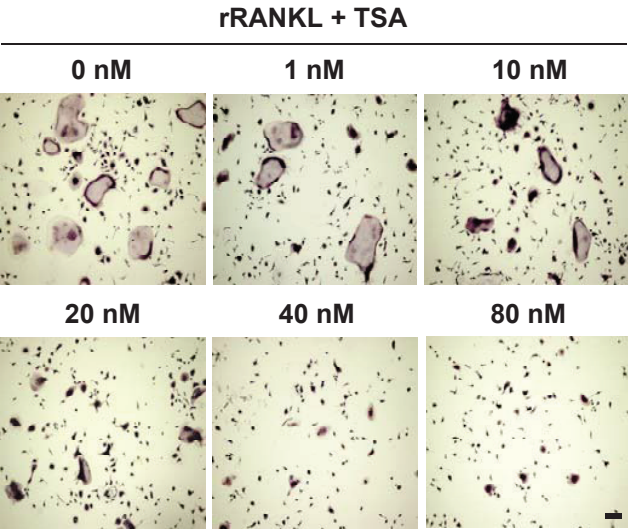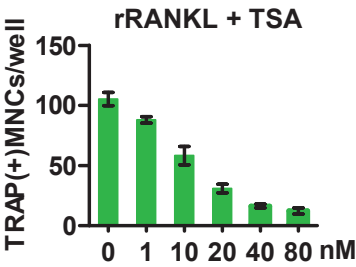

b

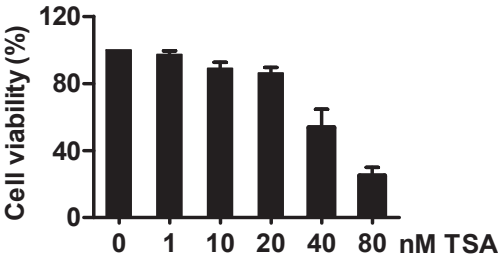

Supplement: Supplementary file 2 — Additional file 2. Effects of increasing concentration of TSA on OCP cell viability and differentiation. a OCP-induced cells were treated with the indicated concentrations of TSA for 5 days and subjected to TRAP staining analysis. b OCP cells were treated with TSA as in (a), and their viability was scored by MTT assay. [file 13072_2019_270_MOESM2_ESM.pdf]

Additional file 3

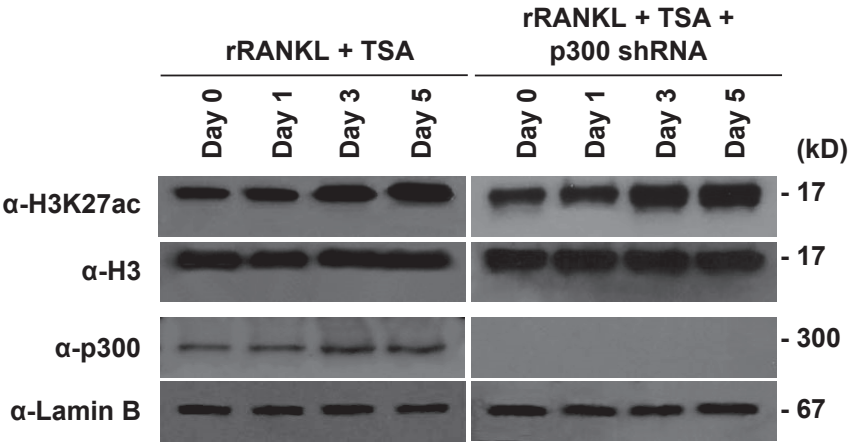

Supplement: Supplementary file 3 — Additional file 3. Analysis of effects of p300 knockdown on H3K27ac in TSA-treated, OCP-induced cells. Mock-depleted or p300-depleted OCP-induced cells were cultured for 0, 1, 3, 5 days in the presence of TSA, and chromatins and nuclear lysates were analyzed by Western blotting with H3K27ac, H3, p300 and Lamin B antibodies. [file 13072_2019_270_MOESM3_ESM.pdf]
